# Stress Fractures in Military Recruits: Current Concepts, Risk Stratification, Prevention, Evidence-Based Management, and Future Directions

**DOI:** 10.7759/cureus.117517

**Published:** 2026-10-05

**Authors:** Mohammed Al-Rumaih, Waleed Al-Hoshan, Mansour Al-Jahny, Talal Al-Malki, Abdulrahman Al-Otaibi, Meshari Al-Shayie, Abdulmajeed Al-Wayil, Salman Al-Harbi

**Affiliations:** 1 Department of Orthopaedic Surgery, Security Forces Hospital, Riyadh, SAU

**Keywords:** injury prevention, military stress fracture, rehabilitation, return to duty, risk factors

## Abstract

Stress fracture is a major cause of morbidity, training loss, retarded graduation, and military loss in recruits where there is an abrupt increase in repetitive mechanical loading. This narrative review synthesized up-to-date data on mechanobiology, risk factors, risk stratification, clinical signs and symptoms, diagnosis, prevention, management, rehabilitation, return to duty, and future directions for stress fracture in military populations. A literature search in PubMed/MEDLINE and Google Scholar identified 35 English-language studies used in the final narrative synthesis.

Existing data indicate a load-capacity model involving dynamic relationships among external mechanical dose, local skeletal strain, and biological repair capacity. Factors contributing to susceptibility are a history of stress fracture, being female, age, menstrual malfunction, pre-entry conditioning, poor body composition, lack of vitamin D, exposure to medication, and site-specific biomechanics. In cases of negative radiographs and a high rate of clinical suspicion, such as activity-related and localized early symptoms, advanced imaging, including MRI, is the choice. Implement site-specific management, with greater protection and surgical attention for high-risk fractures. Rehabilitation should depend on pain-free work, strength recovery, progressive exposure to impact, and signs of healing rather than time.

Prevention should focus on pre-entry conditioning, gradual training loads, adequate rest, adequate nutrition, and early symptom reporting. Future studies should test dynamic prediction models that integrate skeletal imaging, load measures from wearables, and known fracture outcomes to inform prevention and safe return to military service at the individual level.

## Introduction and background

Stress fractures are fatigue-related injuries to the skeleton caused by repetitive submaximal mechanical loading of normal bone at a magnitude, frequency, or duration that overloads the bone's ability to repair [1]. Repeated loading at the tissue level results in microcracks, osteocyte damage, and the initiation of localized remodeling. Osteoclastic resorption removes fatigue-damaged bone, but it only temporarily increases bone porosity, whereas osteoblastic bone formation is much slower to restore mechanical strength [2,3]. Repetitive loading without repair can form microcracks that combine and reduce cortical stiffness and fatigue resistance until a macroscopic crack forms [4]. Exercise thus creates a physiological paradox: mechanical loading is appropriate and promotes adaptive bone formation and skeletal strength, whereas excessive repetitive loading with insufficient relaxation leads to fatigue damage and stress fracture [2,5].

Military recruits are a particularly vulnerable population because entering military service requires a rapid transition from a wide spectrum of civilian activity levels to uniform, repetitive, and sometimes unknown mechanical loading [6]. Exercises such as running, marching, drill, jumping, obstacle-course exercise, and sustained weight-bearing and load carriage cause excessive lower-extremity loading that can precede sufficient skeletal maturation [1,6]. The average number of steps/day in military training is about 15,000, although individual exposure to it varies widely [6]. Notably, fundamental combat training is also osteogenic and possibly harmful. Research indicates that training enhances tibial density and microarchitectural factors, signaling adaptive bone development that requires time and can be overcharged if mechanical demand surges suddenly [7, 8]. This balance of skeletal loading and recovery can also be disrupted by inadequate energy availability, sleep disruption, psychological stress, and nutritional factors [4,9].

The clinical and operational burden is high. Bone stress injuries are estimated to affect 5%-10% of military personnel and are more common in women [4,10]. In a survey of U.S. Army recruits, 14,155 stress fractures were identified. The diagnosis rate was highest during weeks 3-4, followed by a plateau during weeks 5-8 and remaining high after 20 weeks. Women were found to be at risk 4.14 times as often as men [11]. Another study found that 0.5% of recruits in the U.S. Navy had manifested lower extremity symptomatic stress fractures, and the median loss was 56 training days [12]. Female sex, insufficient pre-training physical exercise, low body mass, prior stress fracture, menstrual dysfunction, low levels of vitamin D, and phases of highest initial physical loading are becoming relevant risk factors. However, injury is not caused by any single factor [1,10,13,14].

Stress fractures in military recruits are of interest since, with prompt detection and appropriate measures, training time wastage, medical dropout, frequent stress fractures, and chronic functional impairment can be reduced, and safe recovery to military service can be achieved [1,12,15]. This review seeks to summarize the available current data on stress fractures in military recruits with specific emphasis on risk factors, prevention, diagnosis, treatment, return to duty, and future directions.

## Review

Methodology

A structured literature search was conducted to identify published evidence on stress fractures among military recruits and personnel. PubMed/MEDLINE and Google Scholar were searched for peer-reviewed articles published from January 2018 to September 2026. The search focused on stress fractures, risk factors, prevention, diagnosis, treatment, rehabilitation, and clinical outcomes in military populations.

The PubMed search strategy was: ("Stress Fractures" OR "stress fracture") AND ("Military Personnel" OR "military" OR "recruit" OR "cadet" OR "soldier") AND ("risk" OR "prevention" OR "diagnosis" OR "treatment" OR "rehabilitation"). Similar combinations of relevant keywords were used in Google Scholar.

Studies were included if they were published in English between 2018 and 2026 and involved active-duty military personnel, recruits, cadets, or military trainees. Eligible studies addressed the epidemiology, risk factors, prevention, diagnosis, treatment, rehabilitation, or outcomes of stress fractures. Randomized controlled trials, cohort studies, case-control studies, cross-sectional studies, systematic reviews, and meta-analyses were considered eligible. Studies published before 2018, studies involving only non-military populations, and studies that did not specifically address stress fractures were excluded.

The study-selection process followed a stepwise screening approach. A total of 686 records were initially identified through database searching (PubMed/MEDLINE: *n* = 312; Google Scholar: *n* = 374). Before screening, 142 duplicate records were identified and removed, leaving 544 unique records for preliminary screening. During title and abstract screening, 448 records were excluded for failing to meet basic selection criteria (e.g., non-military populations, non-English publications, editorial letters, or topics unrelated to bone stress injuries). The remaining 96 full-text articles were retrieved and rigorously evaluated against the predefined inclusion and exclusion criteria. Of these, 61 full-text articles were excluded for specific, documented reasons: non-military or civilian-only study populations (*n* = 22); a broad musculoskeletal injury focus without specific data on stress fractures (*n* = 18); ineligible publication designs, such as narrative reviews, opinion pieces, or conference abstracts (*n* = 12); publication outside the January 2018-September 2026 date range (*n* = 6); and non-English language (*n* = 3). Following full-text assessment, 35 studies met all eligibility criteria and were included in this review (Figure 1).

**Figure 1:**
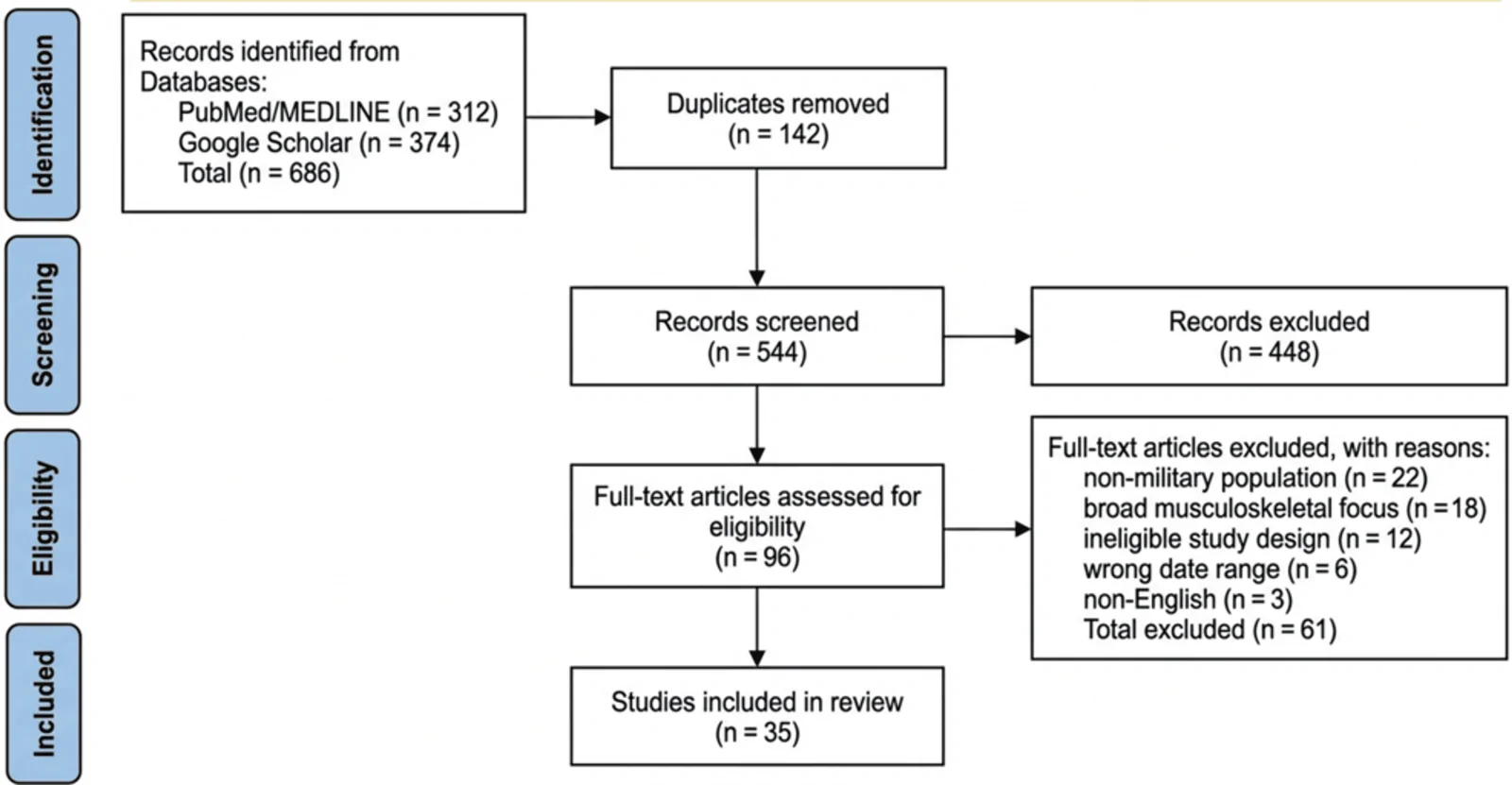
PRISMA flowchart showing the selection and inclusion of studies. PRISMA, Preferred Reporting Items for Systematic Reviews and Meta-Analyses

Results and discussion

Mechanobiology and Pathophysiology

A stress fracture is a fatigue failure of structurally normal bone that occurs when microdamage accumulates faster than skeletal modeling and remodeling can repair it [3]. A fatigue stress fracture occurs when mechanical stress on the bone exceeds the capacity of normal bone to repair and adapt. Risk therefore reflects a dynamic interaction among external dose, internal tissue strain, and biological repair [3,16].

The load-capacity mismatch: The external mechanical dose is not the cumulative skeletal stimulus resulting from any single training variable but results from the magnitude, frequency, rate, direction, and number of loading cycles [16,17]. In military recruits, this dose is created alongside running mileage, marching distances, drill, obstacle courses, and load carriage. At the same time, terrain, footwear, and recovery intervals affect how external loads are transmitted to the skeleton [2]. Running mechanics also affect this dose. A finite-element analysis study concluded that reducing flight time during running reduced peak tibial-fibular strain by 17% and the volume of highly strained bone by 35% [18].

Biological capacity is another factor that indicates whether the microscopic damage accumulated during loading bouts can be repaired [16]. Endocrine disturbances during long-term military training may alter this capacity. During strenuous military training, a study showed progressive activation of the hypothalamus-pituitary-adrenal axis and increased suppression of reproductive-axis activity in women [19]. Micronutrient status also influences skeletal capacity. A meta-analysis reported that serum 25-hydroxyvitamin-D levels in military individuals with stress fractures were lower than in controls by -7.33 nmol/L (95% CI, -9.54 to -5.12 nmol/L) in the military subgroup [20]. Nonetheless, a randomized trial of British Army male recruits revealed that oral supplementation or simulated sunlight to reach vitamin D adequacy did not alter structural adaptation of the tibia, indicating that vitamin D status is only a part of a much more complex mechanobiological system [21].

Microdamage and targeted remodeling: Osteocytes, embedded in the mineralized matrix, accommodate mechanical loading by sensing deformation and fluid flow through the lacunar-canalicular network and translating these into biochemical signals [22]. Recent mechanobiology findings indicate that integrins, ion channels, and intracellular effectors such as Yes-associated protein (YAP) and transcriptional coactivator with PDZ-binding motif (TAZ) and Wnt/β-catenin facilitate the translation of mechanical stimuli into osteogenic responses [23]. When the mineralized matrix is not able to sustain the recurring strain, it is damaged at the microscopic level and progressively impairs the mechanical competence of the local tissue [24]. When the osteocyte signaling changes, remodeling takes place, recruiting and activating osteoclasts, which then resorb the damaged tissue and replace it with osteoblasts [25]. This remodeling creates a temporal problem, with osteoclastic resorption being replaced by a duration of local structural competence boosted by osteoid deposition before it fully mineralizes [24].

The military-training *vulnerability window*: Military studies provide strong evidence that early training can simultaneously stimulate beneficial formation and a transient period of cortical vulnerability [8]. In 91 female army recruits undergoing eight weeks of basic combat training, structural indices of trabecular structures and estimated bone strength improved, while cortical vBMD and cortical tissue mineral density decreased modestly by around 0.3%-1.0% [8]. This sequence is well supported by a long-term study in the British Army carried out over 44 weeks by O'Leary et al. [25]. Cortical vBMD initially decreased by 0.3% by week 14. In contrast, the relevant sites of trabecular volumetric bone mineral density (vBMD), cortical area, cortical thickness, and estimated failure load increased by 3.0%, 4.8%, 4.0%, and 2.5%, respectively, after training [25].

Newer data indicate that this adaptive response varies widely within the population and is not universal across a recruit cohort. Kargl et al. also reported that among marine officer candidates, those with the highest tibial strength also showed the highest osteogenic activity of circulating factors in cell-culture experiments (higher osteoblast mineralization and alkaline phosphatase activity); females were more likely to be classified as non-adapters [26]. The *vulnerability window* is thus not a chronologically defined number of training weeks but rather an individually determined time point in which mechanical demand has increased more rapidly than mature structural adaptation [2,26].

Risk Factors and Risk Stratification

Non-modifiable risk factors: Risk stratification should start by recognizing the recruit's baseline susceptibility. Previous stress fracture is one of the most reproducible predictors. Previous stress fracture and irregular menstruation were also reported as important non-modifiable risk factors, and aging was found to be a risk factor as well [13]. Consistently, Campbell et al. found that older age (in one of 70 military studies) and female sex were risk factors in their systematic review [1]. Sex is one of the strongest population-level predictors. Kardouni et al. identified 14,155 stress fractures in Army soldiers and reported a 4.14-fold higher hazard in females than males [11]. Sex is not likely to be the sole cause of female susceptibility. This association may be mediated, in part, by smaller skeletal dimensions, reduced lean mass, menstrual dysfunction, low energy availability, and endocrine factors [27]. Gifford et al. also demonstrated sex-specific hypothalamo-pituitary-adrenal and reproductive-axis responses to challenging training, suggesting that the hormonal milieu mediates the association rather than sex alone [19]. The incidence of stress fractures has also been related to race/ethnicity, but it is recommended that race be considered an epidemiological marker and not a direct cause, as skeletal geometry, body composition, vitamin D status, and environmental exposures could play a role [13].

Modifiable and potentially modifiable risk factors: Intense training without proper conditioning is the most actionable risk factor. In a study by Pihlajamäki et al., 4,029 male Finns were followed for 11 years, and those who exercised regularly before military service had a lower risk of fatigue fracture than those who did not [28]. Similarly, Campbell et al. found that lack of pre-service exercise (around 7-10 hours per week or ≥3 sessions per week) was an important recruit risk factor [1]. Body composition also affects risk. In Army trainees, Knapik et al. observed a U-shaped relationship in men, with stress fracture risk 27% higher in the lowest decile of predicted body fat than at the median and 15% higher in the highest decile. Risk was 20% greater in the lowest decile in women [14]. Very low body fat and underweight should therefore not be viewed as harmless, especially when associated with high training volume or low energy availability. In addition, a diet high in calcium, potassium, and protein was linked to higher tibial BMC and strength in recruits beginning training, as reported by Nakayama et al. [29].

Vitamin D is the most biochemically supported vitamin today. In their meta-analysis, Moraes et al. found that 25-hydroxyvitamin-D levels were significantly lower in patients with stress fractures than in controls, with a mean difference of -7.33 nmol/L in the military subgroup [20]. Armstrong et al. also reported that increased week-15 25-hydroxyvitamin-D levels correlated with decreased fracture risk in Royal Marines [30]. 

Another modifiable factor is medication exposure. Hughes et al. evaluated 1.26 million soldiers in the United States Army. They determined that overall prescriptions for nonsteroidal anti-inflammatory drugs (NSAIDs) were linked with a 2.9-fold higher rate of stress fractures and a 5.3-fold higher rate during Basic Combat Training [31]. Biomechanics should also be considered site-specific. Dixon et al. linked second metatarsal fractures to a higher-arched foot pattern and lower forefoot abduction, while third metatarsal fractures correlated with later forefoot peak pressure [32]. Risk stratification should therefore not be based on any single screening test. Instead, it should include fixed susceptibility, conditioning, training load, body composition, nutritional status, medication exposure, and site-specific biomechanics. Figure 2 shows a proposed algorithm for stress fracture risk stratification in military recruits.

**Figure 2:**
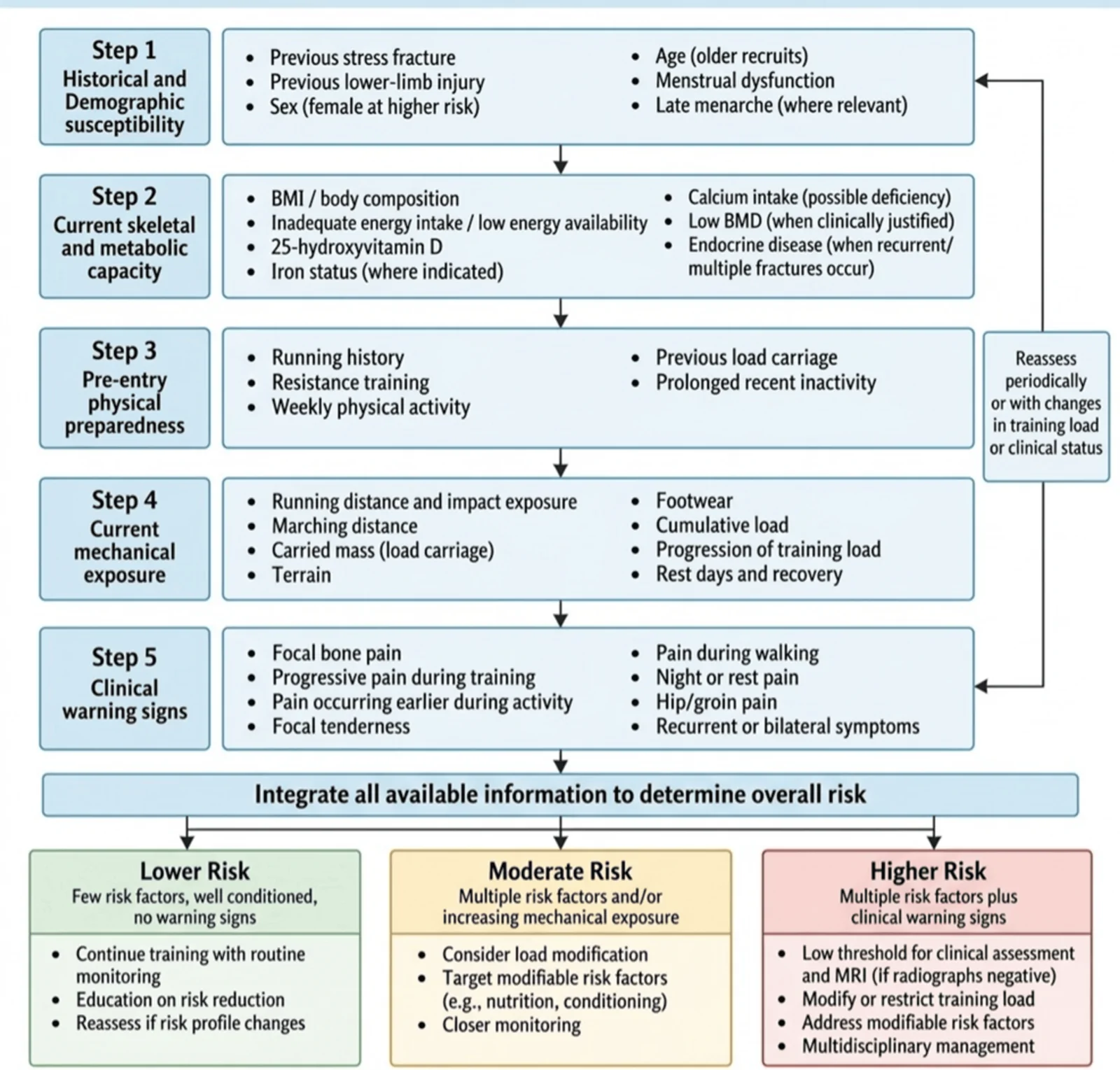
Proposed military stress-fracture risk-stratification algorithm. The image was created by the authors using Affinity Designer Software (version 1.10.6, Serif Europe Ltd (Affinity), Nottingham, United Kingdom).

Clinical Presentation

Stress fractures usually manifest with subtle, focal pain from repetitive loading rather than from a single event and may be relieved by rest but become more frequent with less activity as the structure deteriorates [33]. Early symptoms may follow long-term running, marching, or other impact exercise. Pain then begins during training, occurs earlier and more often with training, and ultimately occurs during normal walking or activities of daily living [34]. Rest or night pain, gait alteration, or inability to bear weight in more severe lesions should indicate a higher-grade or high-risk fracture and not uncomplicated training-related musculoskeletal pain [35].

Physical examination typically shows localized bony tenderness over the painful area and, in some cases, localized swelling; diffuse muscular tenderness is less typical of a structural stress fracture [35]. Presentation varies significantly by location. Femoral-neck stress fractures usually result in atraumatic hip or groin pain that is made worse by weight-bearing and is relieved by rest at first, but delayed diagnosis can lead to displacement [36].

Diagnostic Imaging

Plain radiography: Plain radiography is the most readily available and cost-effective imaging modality and is recommended as the first imaging investigation due to its ability to detect cortical lucency, periosteal new bone, sclerosis, cortical thickening, callus, or a complete fracture [37]. Its main limitation is limited early sensitivity, since radiographic changes may only become apparent after adequate cortical remodeling [38]. Shapiro et al. examined 62,371 military recruits, of whom 3,672 were clinically diagnosed with a stress fracture; of these, only 11.1% were radiographically positive, and there was no significant difference in the diagnostic yield when radiographs were taken prior to or following 21 days [39]. However, a normal early X-ray does not rule out a clinically significant fracture.

Magnetic resonance imaging (MRI): MRI is the preferred advanced modality when radiographs are negative or equivocal, but clinical suspicion remains high, and the American College of Radiology (ACR) criteria identify it as the principal modality for detecting radiographically occult stress fractures [37]. MRI shows periosteal and marrow changes, cortical damage, and a low-signal fracture line and allows evaluation of adjacent soft tissues without ionizing radiation [40]. Bernstein et al. noted that MRI morphology in femoral-neck stress fractures can guide treatment decisions and that MRI is better at identifying and classifying high-risk incomplete lesions [36].

Computed tomography (CT): CT offers better visualization of the morphology of the fracture lines and of the cortical architecture and is useful if MRI findings are indeterminate, for complex high-risk sites, and for the analysis of healing, sclerosis, delayed union, and nonunion [38]. This is particularly relevant in fractures of the navicular and proximal fifth metatarsal, where detailed cortical assessment can help direct treatment [41]. However, CT is less appropriate than MRI for detecting the earliest marrow-dominant abnormalities.

Bone scintigraphy: Bone scintigraphy is very sensitive for detecting increased bone turnover and may show multifocal skeletal involvement, but it is less anatomically specific and involves ionizing radiation in recruits. Shapiro et al. found 49.7% of bone scans to be positive and 49.2% of positive scans to show fractures of more than one bone in the military cohort [39]. As a result, its diagnostic role has diminished at sites where MRI is readily available, but it remains useful in specific situations where MRI is unavailable or contraindicated [37].

Evidence-Based Management

Immediate management: Initial management should begin with immediately stopping the provoking activity, followed by classification based on anatomical location, fracture morphology, and the risk of propagation, displacement, delayed union, or nonunion [33,34]. Activity modification and symptom-guided weight bearing are often adequate for low-risk fractures, while femoral-neck, navicular, anterior-tibial-cortex, and certain fifth-metatarsal fractures are more amenable to more protective management [35]. Shaw et al. recommended a low threshold for MRI and weight restriction in cases of femoral-neck stress fracture. In most cases, compression-sided fractures with an area less than 50% of the femoral neck width can be treated nonoperatively, while tension-sided, complete, progressing, or ≥50%-width fractures should generally be considered for surgical intervention [42].

Rehabilitation and return to training/return to duty: Rehabilitation should be based on criteria rather than time alone. A practical progression starts with impact-loading protection and subsequent cardiovascular conditioning from non-impact exercises, then restoring pain-free range of motion, calf/hip/lower-extremity strength, and functional loading capacity. Schundler et al. reported high return-to-sport rates following both non-operative and operative treatment, although some groups had a non-operative failure rate exceeding 25%, highlighting the importance of clinical and imaging reassessment if symptoms persist [43].

If walking and everyday activities are pain-free, impact can be gradually reintroduced via walking, brisk walking, walk-jog intervals, and continuous running, progressing distance first, then speed and intensity. George et al. found that key factors that must be met before returning to running include pain-free walking, resolution of bony tenderness, restored strength and functional loading capacity, and radiological healing for high-risk lesions [44]. Military-specific reloading should then be followed by restoration of marching, boots, uneven terrain, load carriage, drills, and occupational tasks before unrestricted duty. For high-risk fractures, more evidence of union is required, such as the recent consensus recommendations in navicular surgery, which include protected non-weight-bearing and structured rehabilitation before unrestricted sport [45]. Figure 3 summarizes the rehabilitation stages in the management of military stress fractures.

**Figure 3:**
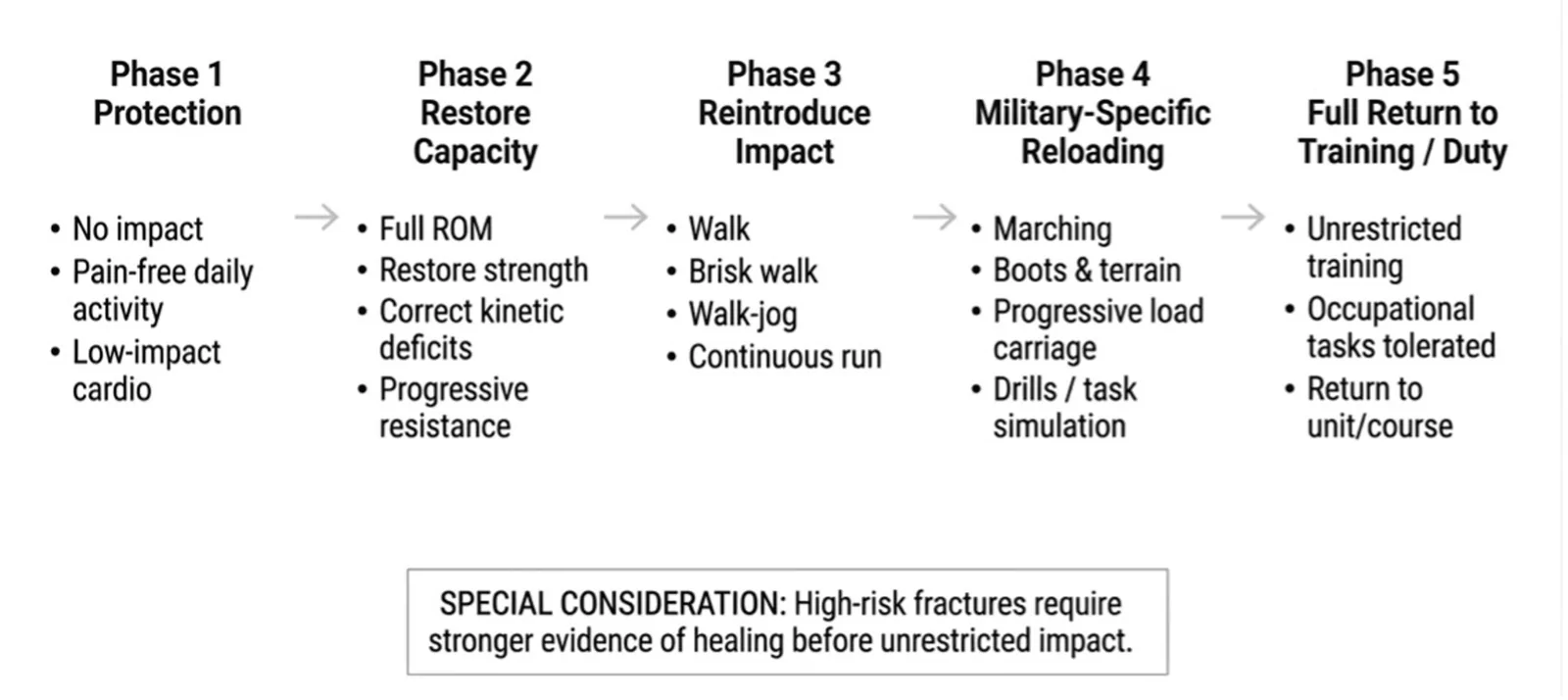
Criteria-based rehabilitation and return-to-duty pathway. The image was created by the authors using Affinity Designer Software (version 1.10.6, Serif Europe Ltd (Affinity), Nottingham, United Kingdom).

Return-to-duty outcomes: Fracture healing and return to military duty are not equivalent outcomes. Return-to-duty reporting must differentiate radiographic union, pain-free walking, return to running, return to training, original course completion or recertification, full unrestricted duty, permanent restriction, and military separation. However, Griffis et al. examined 53 active-duty soldiers who had surgery for incomplete femoral-neck stress fracture and had a 67% nonreturn to duty despite fracture stabilization with fixation to prevent progression to complete or displaced fracture [46]. Shaw et al. also documented 58% military separation for 57 surgically treated basic-trainee femoral-neck stress fractures, which highlights the significant occupational implications of this high-risk injury [47]. Biological healing, functional recovery, and the physical demands of the military job should form the basis of return to duty, rather than fracture union alone.

Preventive Measures

The prevention of stress fractures in military recruits should be implemented prior to exposure to rigorous training, and the aim should be to enhance the capacity of the skeleton to sustain the mechanical load and not to overload it easily. Pihlajamäki et al. reported that the risk of fatigue fracture was significantly reduced in Finnish conscripts who exercised more than twice per week before service [28]. Campbell et al. also found that limited pre-service exercise and periods of highest physical loading were important recruit-specific risk factors [1]. Training should therefore be gradual, beginning with running and marching, followed by resistance work and load carriage, and with proper rest periods between high-impact sessions. This is especially critical during the initial stages of training. Kardouni et al. reported 14,155 stress fractures in 701,027 U.S. Army soldiers, with the incidence rates rising from weeks 3-4 and peaking around weeks 5-8 [11].

Mechanical conditioning should be complemented with sufficient energy and nutrient availability. O'Leary et al. showed that during strenuous military training, bone-specific alkaline phosphatase increased with additional energy, but they did not determine the impact on stress fracture incidence [48]. Nakayama et al. observed that a diet higher in calcium, potassium, and protein was correlated with higher tibial mineral content and estimated strength in recruits entering training [29]. Moraes et al. reported reduced 25-hydroxyvitamin D levels in soldiers with stress fractures, supporting assessment and correction of low vitamin D levels, but not indiscriminate supplementation [20]. This is a safe measure, as Gaffney-Stomberg et al. demonstrated lower biochemical bone-resorption responses when taking 1,000 mg of calcium and 1,000 IU of vitamin D daily, but no alterations in tibial microarchitecture in simple training [49].

Future Directions

Future research must redirect its attention towards dynamic, personalized prediction of stress fracture risk in the context of military training. Advanced high-resolution peripheral quantitative CT for skeletal assessment, cortical geometry, and finite-element estimates of bone strength with real-time mechanical exposure might more accurately describe the relationship between external load and local skeletal strain [16]. Wearable technologies could also measure the number of steps, impact magnitude, running/marching volume, load carried, and recovery, allowing risk to be recalculated as training exposure varies. Biomarker research should also include a panel of markers such as 25-hydroxyvitamin D, bone-formation and resorption markers, iron status, endocrine responses, and energy-availability indices [50]. Vitamin D receptor variations are genetic factors that can alter susceptibility but are not yet ready for clinical screening [30]. Finally, artificial intelligence models would need to include demographics, prior fracture, fitness, skeletal geometry, nutrition, biomarkers, symptoms, and load measured with wearables, but future models would require prospective validation using true stress fracture as the primary outcome, rather than MRI changes alone.

## Conclusions

Stress fractures continue to be a major cause of training disruption and loss of military personnel, and they develop due to disproportionate repetitive mechanical loading, skeletal strain, and biological repair. Effective management relies on timely identification, anatomical risk grading, appropriate imaging, location-based treatment, and criterion-based rehabilitation before returning to work. Prevention measures should focus on pre-entry conditioning, gradual training loads, sufficient recovery, adequate energy and nutrient intake, and early reporting of focal bone pain. Future research should prospectively test dynamic risk-stratification models that combine all of these factors, including skeletal geometry, biomarkers, exposure information from wearable devices, and clinical symptoms, and use stress fracture and clinically relevant RTD factors as primary outcomes across a variety of military recruit cohorts.
